# Congenital Absence of the Left Circumflex Artery Presenting With Inferoposterior Wall Myocardial Infarction Due to Stenosis of the Super Dominant Right Coronary Artery: A Rare Case

**DOI:** 10.7759/cureus.46709

**Published:** 2023-10-09

**Authors:** Kanishk V Khurana, Aayushi Singh, Tarun Rao, Saket Toshniwal, Sourya Acharya, Gajendra Agrawal, Anuj Chaturvedi

**Affiliations:** 1 Medical School, Jawaharlal Nehru Medical College, Datta Meghe Institute of Higher Education and Research, Wardha, IND; 2 Medicine, Jawaharlal Nehru Medical College, Datta Meghe Institute of Higher Education and Research, Wardha, IND; 3 Cardiology, Jawaharlal Nehru Medical College, Datta Meghe Institute of Higher Education and Research, Wardha, IND

**Keywords:** acute myocardial infarction, steal phenomenon, coronary angiography, super dominant rca, av groove, congenital absence of lcx

## Abstract

The primary coronary arteries are the right coronary artery (RCA), the left main coronary artery (LMCA), which bifurcate into the left anterior descending artery (LAD), and the left circumflex artery (LCX), arising from the right coronary sinus and left coronary sinus, respectively. The congenital agenesis of LCX is a very unusual anomaly caused by the inability of LCX to form in the atrioventricular (AV) groove. This condition is usually accompanied by the presence of a large, dominant RCA that supplies its own territory and that of LCX, i.e., the inferior, posterior, and lateral walls. This anomaly is generally detected incidentally during coronary angiography. This condition usually does not manifest as a major cardiovascular event and mildly presents as chest pain upon exertion. The chest pain is vastly attributed to ischemia in the RCA territory, as this "super dominant" vessel majorly directs its supply to the LCX territory for compensation. This is known as the steal phenomenon. In this paper, we discuss a case of a 61-year-old female who came to the ED with the chief complaint of acutely radiating chest pain for five hours and was diagnosed as a case of acute myocardial infarction of the inferior and posterior walls. Coronary angiography revealed 90% stenosis of the RCA and a congenital absence of LCX, which has a significantly low prevalence.

## Introduction

The aortic sinus gives rise to the coronary arteries that supply the heart. The sinus of Valsalva (aortic sinus) is divided into three parts: the right, or anterior coronary sinus; the left, or posterior coronary sinus; and the non-coronary sinus. The right coronary artery (RCA) arises from the right coronary sinus and gives blood supply to the right chambers of the heart, the sinoatrial node, the atrioventricular node, and a portion of the left ventricle. The left main coronary artery (LMCA) originates from the left coronary sinus and divides into the left anterior descending artery (LAD) and left circumflex artery (LCX), which supply the left atrium and the left ventricle. [1]. The coronary arteries can be associated with several congenital anomalies that are usually detected accidentally during coronary angiography [2]. Their prevalence lies between 0.6% and 1.3%, according to various studies [3]. The most common coronary artery anomaly is a 'split RCA' or 'double RCA' with an incidence of 1.23%. Some other anomalies include duplication of LAD, duplication of LCX, absence of LMCA, absence of LCX, LMCA atresia, congenital hypoplasia of RCA and LCX, coronary fistulas, and arteriovenous malformations. Out of these, the congenital absence of LCX has a very low prevalence of only about 0.003% to 0.067% [4]. It is a very uncommon anatomical defect of the coronary arteries that is frequently coupled with a super dominant RCA [5]. The RCA passes through the cardiac crux to lie in the left AV groove and extends to supply the posterolateral wall of the heart, thus covering the LCX territory along with its own, i.e., the inferior wall [4]. In 59% of cases, these patients commonly presented with a history of chest pain on exertion, whose etiology is best described by the 'steal phenomenon' and was documented in a case report and review of the literature published by Fugar et al. in 2017 [6]. However, in extremely rare situations, these patients can present with acute myocardial infarction and ECG abnormalities, as seen in our case report of a 61-year-old female that has been presented below [7].

## Case presentation

**Figure 1:**
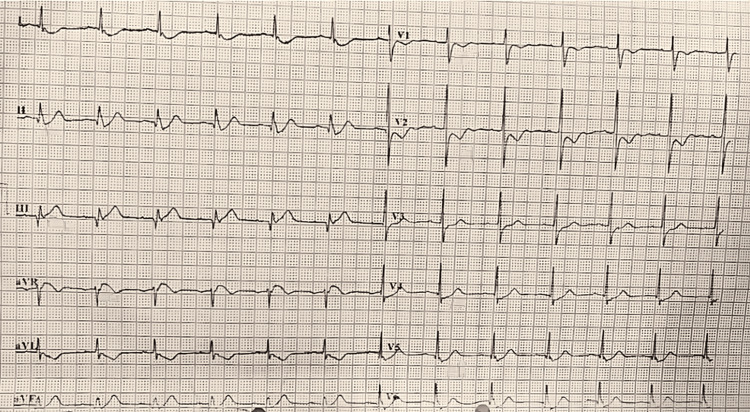
The electrocardiogram shows an inferior and posterior wall myocardial infarction.

A 61-year-old female with no history of any comorbidity reached the ED with chief complaints of diffuse chest pain for five hours. The chest pain radiated to the left arm and the shoulders bilaterally. There was no history of dyspnea, syncope, palpitations, nausea, vomiting, or fever. The patient was referred to this hospital by her primary care physician, where she was diagnosed with an inferior and posterior wall myocardial infarction based on the standard 12-lead ECG recording and was given a loading dose of aspirin 300 mg, clopidogrel 600 mg, atorvastatin 40 mg, and injection enoxaparin 0.6 mg subcutaneously. Upon general examination in the hospital, her vital signs showed a pulse rate of 56 beats per minute, a respiratory rate of 14 breaths per minute, a spO2 of 97%, and a blood pressure of 96/60 mmHg. The patient was given 500 ml of normal saline to stabilize the blood pressure. Further, a cardiovascular examination was performed, wherein the first and second heart sounds were heard normally and no murmurs were found. An ECG was done again at the hospital, which showed ST-segment elevation in leads III, avF, and avR with reciprocal ST depression and T-wave inversion in leads V1 and V2, confirming the diagnosis of an inferior and posterior wall myocardial infarction (Figure 1).

**Figure 2:**
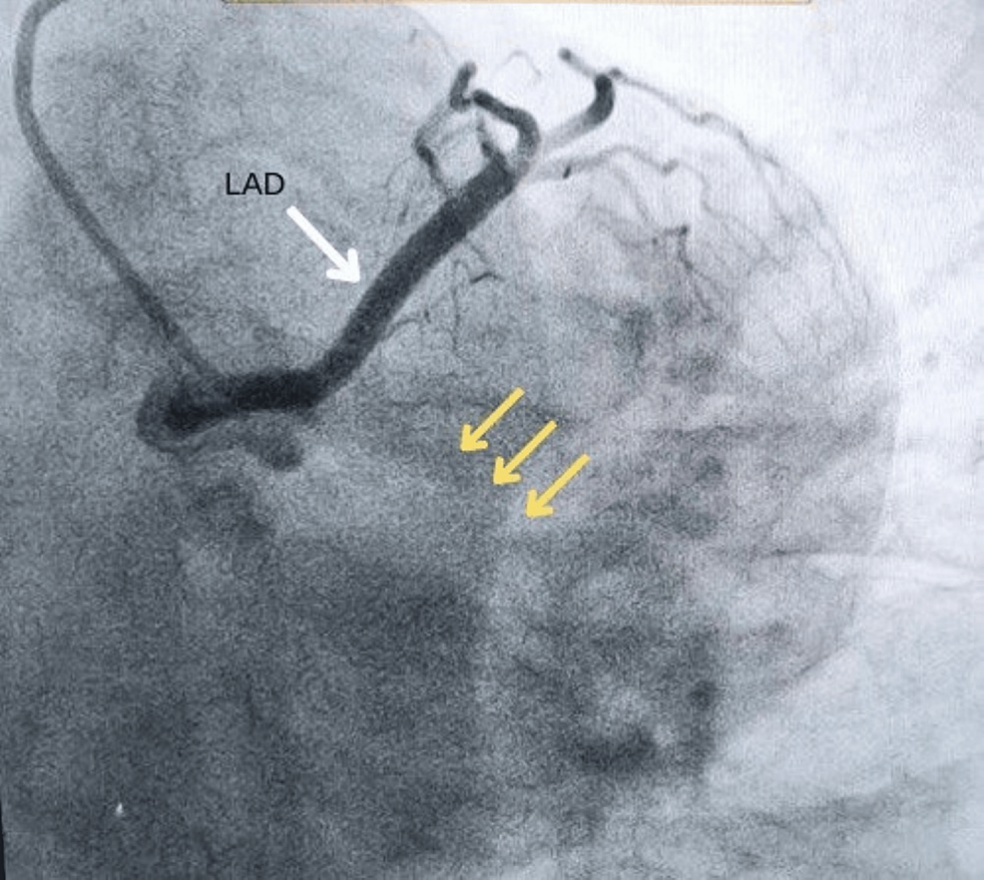
AP-caudal view showing LAD (white arrow) and absent LCX (yellow arrows); no separate ostium for LCX is visible. AP: anteroposterior, LAD: left anterior descending coronary artery, LCX: left circumflex coronary artery

**Figure 3:**
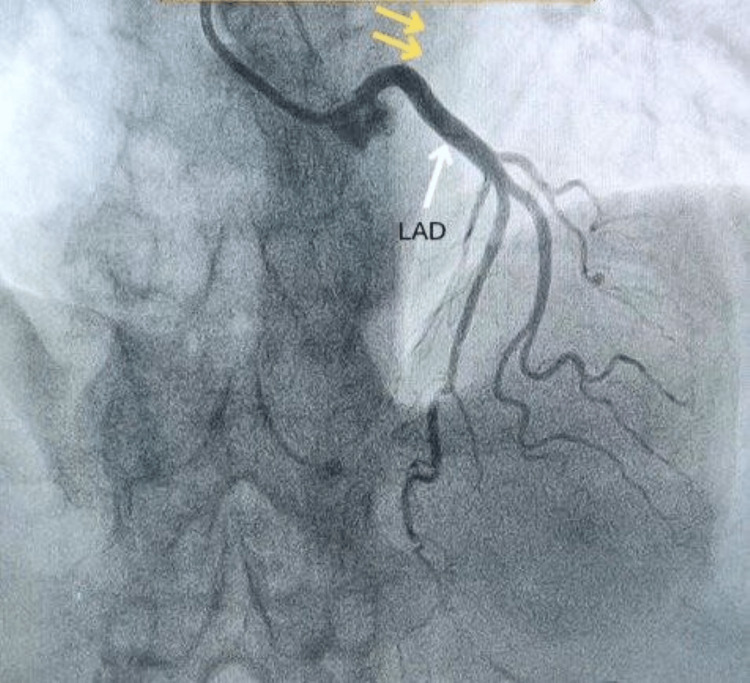
LAO-cranial view showing LAD and its branches (white arrow) with an absent LCX (yellow arrows) LAO: left anterior oblique, LAD: left anterior descending coronary artery, LCX: left circumflex coronary artery

**Figure 4:**
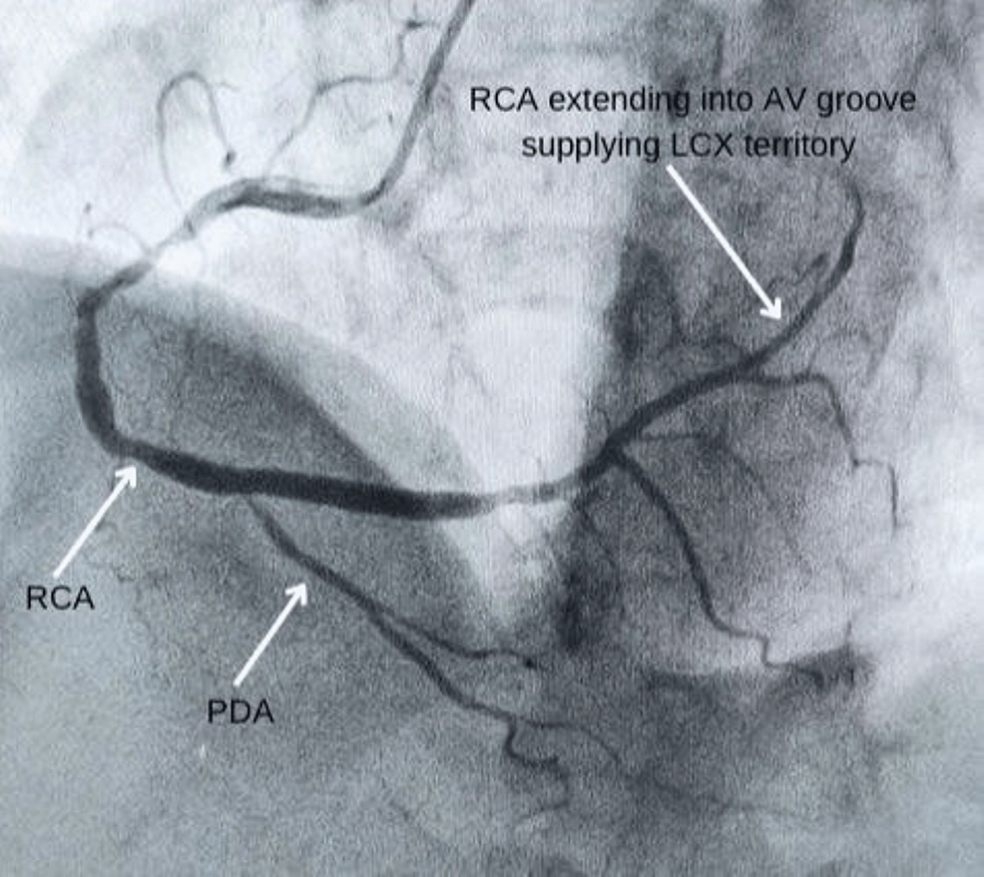
LAO-cranial view showing a super dominant RCA, its extent, and PDA LAO: left anterior oblique, RCA: right coronary artery, PDA: posterior descending coronary artery

**Figure 5:**
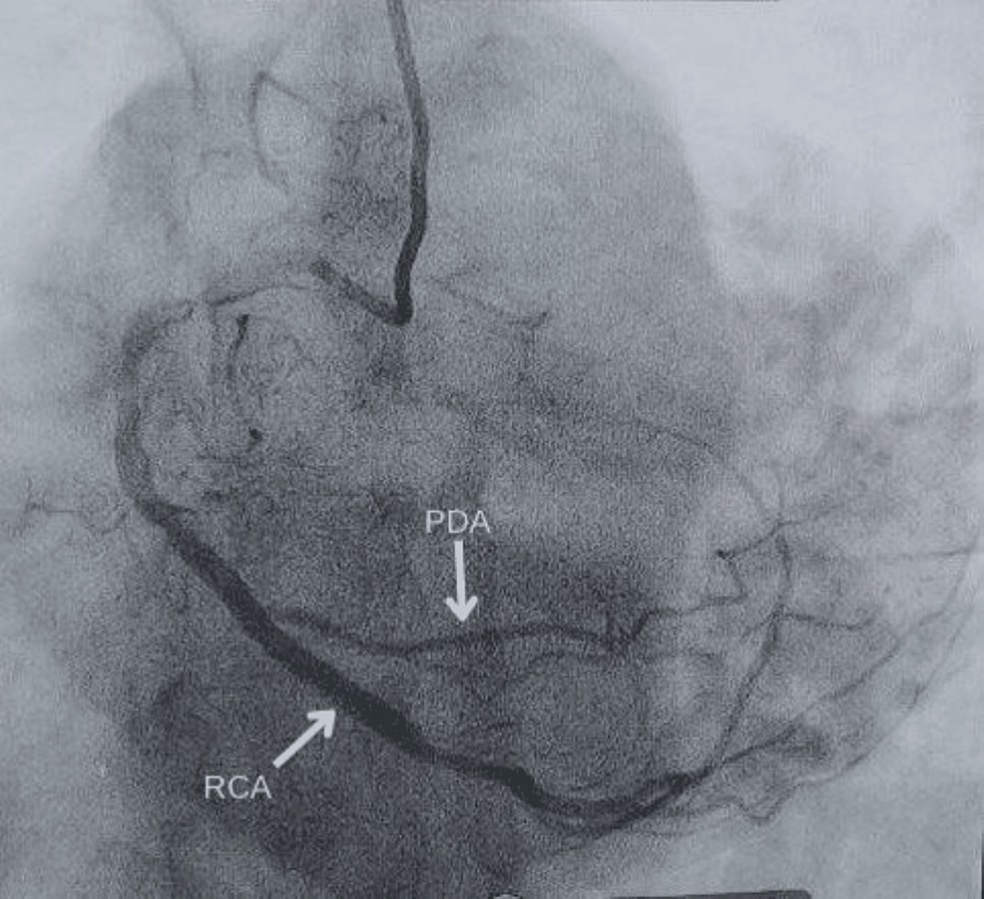
PA-cranial view showing a superdominant RCA supplying LCX territory and PDA PA: posteroanterior; RCA: right coronary artery; LCX: left circumflex coronary artery; PDA: posterior descending coronary artery

Along with ECG, the necessary investigations for the estimation of cardiac biomarkers were done, which showed creatine kinase-myoglobin binding (CK-MB) and high-sensitivity cardiac troponin I (hs-cTnI) to be 79 IU/L (normal range: 5-25 IU/L) and 501 ng/L (normal value: below 14 ng/L), respectively. A transthoracic echocardiogram showed mild mitral regurgitation (MR) with inferior, lateral, and septal wall hypokinesia, grade I diastolic dysfunction, and a left ventricular ejection fraction (LVEF) reduced to 45%. Coronary angiography was performed with a diagnostic catheter (5F TIG) via the right radial artery using non-ionic Omnipaque contrast, which revealed 90% stenosis in the proximal RCA, which appeared to be a large super-dominant artery extending from the right aortic sinus to the AV groove and then the LCX territory. Further, the LCX was neither visible in its distinguished territory nor was any distinct ostium seen for it in the posterior aortic sinus. The images of the coronary angiography have been shown below in Figures 2-5.

Further, within one hour of admission, a percutaneous transluminal coronary angioplasty (PTCA) was conducted for the stenosed super dominant RCA with a Racer CC plus stent (3.50 mm x 48 mm), and following this, standard post-PTCA medical management was provided, which included atorvastatin 40 mg H/S, aspirin 75 mg once daily (OD), and clopidogrel 75 mg OD. Since the patient developed pulmonary congestion later on after MI, furosemide 20 mg twice a day (BD) was also prescribed. Hence, the diagnosis was made as a rare case of congenital LCX absence with a large super dominant proximally stenosed RCA presenting with acute myocardial infarction of the inferoposterior wall.

## Discussion

Congenital absence of LCX, like any other congenital coronary anomaly, is an incidental finding during a coronary angiography procedure with a prevalence of only about 0.003% to 0.067% [4]. In a normal individual, LCX primarily arises as a branch of the LMCA that traverses to the left and enters the coronary sulcus or the AV groove, after which it crosses the diaphragmatic cardiac surface and generally terminates before arriving at the posterior interventricular sulcus [4]. However, in very rare circumstances, the LCX fails to develop embryologically in the AV groove. During a coronary angiography study, if the LCX is not visible in its usual location, it could indicate either complete 100% stenosis due to a large atheromatous plaque or a developmental failure of the LCX. Some other possibilities found from the literature available are an anomalous origin of the LCX from the RCA, straight from the left sinus of the Valsalva, or, in very few cases, from the pulmonary artery or the non-coronary sinus [8]. Usually, the RCA and LCX pass through the AV groove in a loop; however, in our case, there was a complete absence of the LCX and its branches, which is a very uncommon occurrence, and the LMCA continued as the LAD alone [9]. In a situation like this, the RCA takes over as the primary vessel, and its distal branches flow further in the opposite direction, supplying the area of the left ventricle, which usually comes under the territory of the LCX. Due to this extension of the RCA, it becomes exceptionally large in size and is hence referred to as a 'super dominant' artery [8]. In this case, the posterior left ventricular branch of the RCA was prominent and tortuous and continued retrogradely in the left atrioventricular groove into the LCX territory. A congenital absence of LCX can be identified in coronary angiography as a lack of filling of the vessel while injecting the contrast dye into the coronary arteries. This lack of filling of the LCX artery during coronary angiography can support two hypotheses: complete congenital agenesis of the LCX or 100% occlusion of the proximal part of the artery. The latter was not the scenario in our patient because a complete blockage at the location of the origin of LCX would have shown a small amount of contrast at the beginning, appearing as a 'stump' on the imaging; however, no such 'stump' was visible in our patient. Also, there were no collateral fillings, and a large superdominant RCA was clearly visualized, which otherwise would not be seen in 100% occlusion of LCX at the site of the ostium [10]. However, cardiac CT angiography (CCTA) is a superior investigation to confirm the finding of the congenital absence of the LCX, as CCTA is an effective tool for describing coronary anomalies since it provides 3D images of the heart that can accurately show the origin, path, and end of the coronary arteries as well as their relationship with both cardiac and non-cardiac structures [5]. The pathophysiology of LCX absence can be best explained by the steal phenomenon, which describes the transient ischemia leading to chest pain upon exertion experienced in this condition as a result of decreased perfusion of the RCA territory, as the RCA majorly supplies blood to the territory of LCX as a compensatory mechanism. This way, the LCX territory' steals' from that of RCA. The symptoms of this condition mimic the chest pain associated with acute coronary syndrome but are not that severe clinically, as they rarely lead to any major catastrophic event [11]. However, our patient had no such history of chest pain on exertion and rather presented with an episode of acute chest pain on rest due to inferoposterior wall myocardial infarction owing to 90% stenosis of the proximal RCA. If someone has a super dominant RCA due to a lack of LCX, then, the stenosis of the RCA typically causes a myocardial infarction in the inferior, posterior, and lateral walls of the heart and should be treated as a two-vessel disease, as the singular RCA is a superdominant artery covering its own as well as LCX territory [12]. Although the absence of an LCX has not been linked to any significant cardiovascular disorders, it is crucial to identify patients with this anomaly before subjecting them to any cardiac surgical procedures. This is because such patients have a higher risk of being wrongly diagnosed during cardiac catheterization procedures due to the 100% occlusion of LCX, which can lead to incorrect treatments being administered. In addition, patients with an absent LCX require extra care during cardiac bypass procedures to prevent accidental ligation or cutting of anomalous vessels and to ensure the accurate placement of grafts for restoring perfusion [13].

## Conclusions

Congenital absence of LCX with superdominant RCA is a rare anomaly, and the presentation of this condition with stenosis of the RCA causing myocardial infarction is another extremely rare scenario. The lack of LCX is generally detected upon coronary angiography and is primarily confirmed by CCTA. In the absence of CCTA, a super dominant RCA, no separate ostium for LCX and no stump at the location of the division of LMCA serve as secondary confirmation. It is very important to identify this condition before coronary artery bypass grafting (CABG) surgeries to prevent accidental ligation or cutting of anomalous vessels and aid in proper graft placement.
